# Amino acids in the cultivation of mammalian cells

**DOI:** 10.1007/s00726-016-2181-8

**Published:** 2016-02-01

**Authors:** Andrew Salazar, Michael Keusgen, Jörg von Hagen

**Affiliations:** Institute of Pharmaceutical Chemistry, University of Marburg, 35032 Marburg, Germany; Biopharm Materials & Technologies R&D, Merck Lifescience, 64293 Darmstadt, Germany

**Keywords:** Amino acid, Bioprocess, Biotechnology, Cell culture media, Media formulation

## Abstract

Amino acids are crucial for the cultivation of mammalian cells. This importance of amino acids was realized soon after the development of the first cell lines, and a solution of a mixture of amino acids has been supplied to cultured cells ever since. The importance of amino acids is further pronounced in chemically defined mammalian cell culture media, making the consideration of their biological and chemical properties necessary. Amino acids concentrations have been traditionally adjusted to their cellular consumption rates. However, since changes in the metabolic equilibrium of amino acids can be caused by changes in extracellular concentrations, metabolomics in conjunction with flux balance analysis is being used in the development of culture media. The study of amino acid transporters is also gaining importance since they control the intracellular concentrations of these molecules and are influenced by conditions in cell culture media. A better understanding of the solubility, stability, dissolution kinetics, and interactions of these molecules is needed for an exploitation of these properties in the development of dry powdered chemically defined media for mammalian cells. Due to the complexity of these mixtures however, this has proven to be challenging. Studying amino acids in mammalian cell culture media will help provide a better understanding of how mammalian cells in culture interact with their environment. It would also provide insight into the chemical behavior of these molecules in solutions of complex mixtures, which is important in the understanding of the contribution of individual amino acids to protein structure.

**Electronic supplementary material:**

The online version of this article (doi:10.1007/s00726-016-2181-8) contains supplementary material, which is available to authorized users.

## Introduction

Amino acids have been recognized as essential nutrients for the in vitro cultivation of cells since the pioneering work of Eagle (1955a, b, c) and Dulbecco and Freeman (1959), who created nutrient supplements containing amino acids and vitamins that allowed for the cultivation of cells in adherent monolayers. Dulbecco’s Modification of Eagles Medium (DMEM) supplemented with serum (Dulbecco and Freeman 1959) continues to be used routinely for the cultivation of cells. However, serum is a possible source of contamination, poses a safety hazard, and varies from batch to batch (Honn et al. 1975; Kane 1983), which would affect reproducibility and can be detrimental to large-scale mammalian cell culture-based processes. Efforts are therefore being made to move towards serum-free cell culture media (CCM) with chemically defined ingredients of non-animal origin.

One of the first chemically defined CCM to be developed was DMEM F12 (van der Valk et al. 2010), which is a 1:1 (v/v) blend of DMEM and Ham’s F12 medium. The balance of amino acids in DMEM and the trace elements supplied by F12 medium allow culturing cells without serum. DMEM F12 is still used as the basis for further development of CCM formulations but continues to be supplied with serum to maintain cell viability and cell density (van der Valk et al. 2010). In the replacement of serum, ingredients such as meat and plant hydrolysates are also being used (Keay 1975; Kim do et al. 2013; Huang et al. 2010; Kim and Lee 2009). Supplementation with serum is not required for growth of cells in these media. However, these hydrolysates are not chemically defined. Chemically defined CCM, where every constituent component has a known chemical identity, is currently being developed and used for the production of peptide therapeutics. Amino acid concentrations are determined by studying the metabolic requirement of the cell clone used. Concentrations are then adjusted to obtain maximum possible yield from a given process (Rouiller et al. 2014; Xing et al. 2011; Altamirano et al. 2004; Carrillo-Cocom et al. 2014). The amino acid sequence of the peptide of interest is monitored for misincorporated amino acids that may arise due to the shortage of an amino acid (Guo et al. 2010; Gramer 2014; Popp et al. 2015). This is resolved by further increasing the concentration of the corresponding amino acid in the media. The final concentration of an amino acid is influenced by its biological roles and is limited by its chemical properties including solubility and stability.

**Fig. 1:**
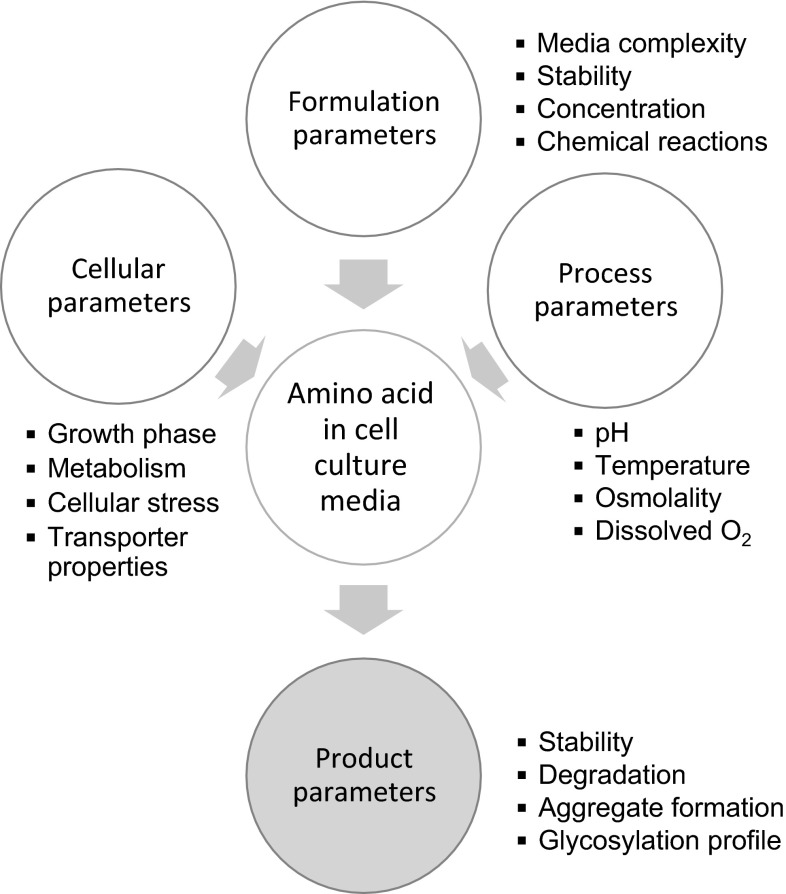
Overview of involvement of amino acids in mammalian cell culture

Thus the availability of amino acids to mammalian cells being cultured depends on their cellular metabolic and transport properties along with the physico-chemical properties of each individual amino acid (Fig. 1). To further optimize the availability of amino acids in CCM, a better understanding of the chemical properties of individual amino acid and their interactions with other components of CCM apart from considering their biological impact in necessary. Previous studies have attempted to address the limitations of stability, where instable l-glutamine was replaced with the dipeptide l-alanyl-l-glutamine and redox active l-cysteine with *N*-acetyl-l-cysteine or *S*-sulfo-l-cysteine (Oh et al. 2005; van der Valk et al. 2010; Hecklau et al. 2016). Solubility limitations have also been addressed using the amino acid derivative phospho-l-tyrosine in place of l-tyrosine (Zimmer et al. 2014).

In large-scale mammalian cell culture, CCM is generated from a powdered mixture. This mixture is dissolved, filter sterilized and stored at 4 °C. This added another layer of complexity making the consideration of additional chemical properties like dissolution kinetics and crystallization essential.

Amino acids are being extensively studied in various different contexts. These studies provide insight into the characteristics of individual molecules, the interactions they are capable of, and their importance to the biology of a cell. This review aims to provide a comprehensive summary of the existing knowledge pertaining to the properties of amino acids in the context of cultivation of mammalian cells.

## Definition of amino acids

Chemically, amino acids are molecules that have either a carboxyl (−COOH) group or acidic character, along with an amino (−NH_2_) group. This definition encompasses a large number of molecules, not all of which are naturally occurring. The twenty proteogenic amino acids and l-cystine (two disulfide bond l-cysteine molecules) as well as l-hydroxyproline are the amino acids most commonly used for the cultivation of mammalian cells (Table 1).

**Table 1:** Amino acid content in cell culture media

	Amino acid	CAS no.	Solubility at 25 °C^b^ (g/kgH_2_O)	Charge at pH 7^b^	Minimum concentration in CCM (g/L)^c^	Maximum concentration in CCM (g/L)^c^
Essential amino acids^a^	l-Arginine	74-79-3	182.60	+(−/+)	0.084	1.331
	l-Cysteine	52-90-4		+/−	0.024	0.123
	l-Cystine	56-89-3	0.11		0.031	0.115
	l-Glutamine	56-85-9	42.00	+/−		
	l-Histidine	71-00-1	43.50	+/−	0.015	0.152
	l-Isoleucine	73-32-5	34.20	+/−	0.050	0.457
	l-Leucine	61-90-5	23.80	+/−	0.050	0.560
	l-Lysine	56-87-1	5.80	+(−/+)	0.000	2.000
	l-Methionine	63-68-3	56.00	+/−	0.015	0.153
	l-Phenylalanine	63-91-2	27.90	+/−	0.015	0.313
	l-Threonine	72-19-5	90.60	+/−	0.020	0.750
	l-Tryptophan	73-22-3	13.20	+/−	0.005	0.080
	l-Tyrosine	60-18-4	0.51	+/−	0.029	0.197
	l-Valine	72-18-4	88.00	+/−	0.020	0.440
Nonessential amino acids^a^	Glycine	56-40-6	239.00	+/−	0.008	0.330
	l-Alanine	56-41-7	166.90	+/−	0.009	0.318
	l-Asparagine	70-47-3	25.10	+/−	0.026	0.589
	l-Aspartic acid	56-84-8	5.04	−	0.013	0.465
	l-Glutamic acid	56-86-0	8.60	−	0.011	0.642
	l-Proline	147-85-3	1625.00	+/−	0.000	0.121
	l-Serine	56-45-1	250.00	+/−	0.030	0.557
Derivatives and dipeptides	Phospho-l-tyrosine	21820-51-9				
	*S*-Sulfo-l-cysteine	1637-71-4				
	l-Alanyl l-tyrosine	3061-88-9				
	l-Alanyl-l-glutamine	39537-23-0				

*+/−* zwitterion, *−* net negative charged, *+*(−/+) net positive charge

^a^Essential amino acids required for cells in culture as determined by Eagle (1955b, c) l-cystine was supplied to these cells as a source of l-cysteine

^b^Haynes (2013)

^c^Landauer (2014)

## Biological roles and impact of amino acids on mammalian cell culture

**Fig. 2:**
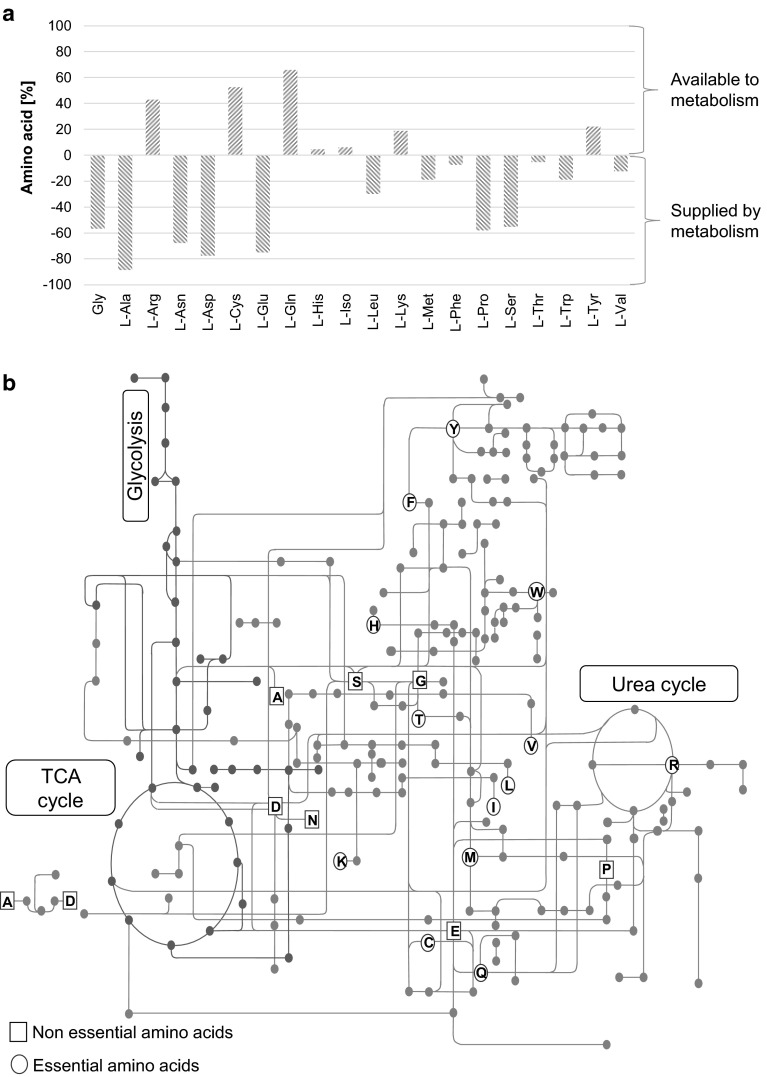
Cellular fate of amino acids in cell culture. **a** Comparison of the amino acid composition of the CHO-K1 proteome with that of DMEM F12. Positive values on the *y* axis indicate the approximate percent of an amino acid supplied by media available to metabolism. Negative values on the *y* axis indicate approximate percent of an amino acid as part of the proteome derived from cellular metabolic synthesis. Protein abundance is not considered. Percentages derived using the ExPASy–ProtParam tool (Table S3) (Consortium U 2008). **b** The possible metabolic pathways of amino acids in the Chinese hamster (*Cricetulus griseus)*. Central carbon metabolism is highlighted in *green*. The *single letter code* is used for the representation of amino acids (Kanehisa et al. 2014)

Amino acids are the basic building blocks of proteins and logically constitute all proteinaceous material of the cell including the cytoskeleton, protein component of enzymes, receptors, and signaling molecules. In addition, amino acids are utilized for the growth and maintenance of cells. A large proportion of CCM-supplied amino acids are diverted to these non-proteogenic pathways that in turn could influence the fate of cells in culture (Fig. 2) (Consortium U 2008).

### Metabolic fates of amino acids

The genetic composition of cells, their gene expression profiles, the cell cycle, and the environment in which cells are present influence the consumption rates and the metabolic flux of amino acids (Vallee et al. 2014; Fomina-Yadlin et al. 2014; Carrillo-Cocom et al. 2014; Yu et al. 2011; Carinhas et al. 2013). Adding amino acids that are generally consumed does not always lead to the improvement of the cell culture process but could lead to undesired effects (Rouiller et al. 2014; Chen and Harcum 2006). Therefore, metabolic profiling and flux analysis combined with stoichiometric analysis of metabolic pathways of cells used in bioprocesses has been performed (Link et al. 2014; Selvarasu et al. 2012; Xing et al. 2011; Chong et al. 2012; Sellick et al. 2011; Orman et al. 2011). With the aim of improving the efficiency of cell culture processes and quality of the molecule of interest, “design of experiments” (DOE) studies have identified amino acids and other components that affect the bioprocess (Rouiller et al. 2014; Kim and Lee 2009; Parampalli et al. 2007; Mandenius and Brundin 2008). The need for the identification of the optimal concentrations of amino acids is particularly important in fed batch and perfusion cultures. Nutrients supplied externally during the culture process in these methods are capable of altering equilibria of metabolic pathways. This can be better explained by considering the role of l-serine and glycine in the tetrahydrofolate (THF) cycle. These two amino acids are involved in the metabolism of nucleic acid precursors through the THF cycle (Amelio et al. 2014; Locasale 2013). In l-serine depleted conditions, however, supplementation with glycine leads to l-serine production. This draws metabolites away from the THF cycle. A slowed THF cycle results in the inhibition of cell proliferation (Labuschagne et al. 2014; Duarte et al. 2014).

Further, essential amino acids are used in the synthesis of non-essential amino acids and other metabolic intermediates (Table 1) (Green et al. 2016). Comparison of the amino acid composition of the proteome (Consortium U 2008) of CHO K1 cells to a chemically defined CCM shows that a major of the proportion of other amino acids in proteins are not derived from CCM but synthesized by cells (Fig. 2). Certain amino acids are available to metabolic pathways at higher concentration than others. These include amino acids involved in the urea cycle and cellular redox metabolism.

### Amino acid transporters

The intracellular availability of amino acids, for proteogenesis or metabolism, is governed by proteins that transport these molecules. Mutations in the genes coding for amino acid transporters lead to diseases such as lysinuric protein intolerance, hyperornithinemia–hyperammonemia–homocitrullinuria, and cystinosis (Torrents et al. 1998; Fiermonte et al. 2003; Kalatzis et al. 2001), which indicates that functional transporters are vital to individual cells and to the entire organism. Animal amino acid transporters are distributed over the cell membrane, inner mitochondrial membrane, and lysosomes (Table 2) (Saier et al. 2014). These transporters have diverse roles, which include redox regulation, connection compartmentalized metabolic pathways and sensing of metabolic states of the cell. They function either as symporters or as antiporters, where two species are transported in the same direction or different direction, respectively.

**Table 2:** Animal amino acid transporter families

TCID	Transporter	Cellular location
1.A.10	The glutamate-gated ion channel family of neurotransmitter receptors	Cell membrane
2.A.3	The amino acid-polyamine-organocation superfamily	Cell membrane
2.A.3.3	The cationic amino acid transporter family	Cell membrane
2.A.3.8	The L-type amino acid transporter family	Cell membrane
2.A.23	The dicarboxylate/amino acid:cation (Na^+^ or H^+^) symporter family	Cell membrane
2.A.29	The mitochondrial carrier transporter family	Inner mitochondrial membrane, perioxysome
2.A.43	The lysosomal cystine transporter family	Cell membrane, lysosome
8.A.9	The rBAT (related to b(0, +) amino acid transporter) transport accessory protein (rBAT) family	Cell membrane

*TCID* transporter classification identification number

Changes in extra cellular metabolites are capable of influencing amino acid transporters. For example, the cystine/glutamate antiporter SLC7A11 (2.A.3.8.5) implicated in the maintenance of the redox state of cells is inhibited by l-glutamate and l-aspartate (Sato et al. 2005; Banjac et al. 2008; Sasaki et al. 2002; Wang et al. 2003). Extracellular concentrations also impact autophagy signaling regulated by amino acids transporters (Wang et al. 2015; Jewell et al. 2015; Hu et al. 2015). Additionally, these transporters are sensitive to metal cation concentrations and pH (Tonazzi and Indiveri 2011; Ruivo et al. 2012; Kalatzis et al. 2001). Changes in expression levels of amino acid transports also need to be considered since, the overexpression of the Ca^2+^ sensitive transporter, SLC25A13, in CHO cells has been shown to increase ATP production in the mitochondria (Lasorsa et al. 2003).

## Chemical properties of amino acids

Amino acids are a diverse set of molecules with a broad range of properties. The amino acids that supplied as part of CCM differ by the –R group bound to the alpha carbon, with the exception of l-proline and l-hydroxyproline (Table S1). The –R group or side chain of the amino acids are varied, ranging from a single hydrogen for glycine to an indole group for l-tryptophan. The side chain influences the molecular weight, hydrophobic/hydrophilic nature, net charge of the molecule, reactive capacity as well as other physico-chemical and biological properties. Although, properties of individual amino acids have been studied extensively, factors influencing mixtures of molecules in solutions are poorly understood. A limited effort has been made to characterize multiple amino acids in solution as would be required for a better understanding of their role in mammalian cell culture.

### Amino acid solubility

To be available to cells, each of the constituent components of CCM needs to be in solution. Thus, the concentration of CCM components, particularly amino acids, is limited by their solubility in a multicomponent solution. Despite sharing common characteristics, the aqueous solubility of amino acids range from $0. 5 4 {\text{g}}/{\text{kg}}_{{{\text{H}}_{ 2} {\text{O}}}}$ for l-tyrosine to $1 2 50 {\text{g}}/{\text{kg}}_{{{\text{H}}_{ 2} {\text{O}}}}$ for l-proline (Haynes 2013). Although the solubility of amino acids increases under basic and acidic conditions (Lee et al. 2013; Tseng et al. 2009), the exploitation of this phenomenon is limited in the development of CCM, because culturing cells requires a pH near neutrality. At this pH, most amino acids are in their least soluble zwitterionic form.

**Fig. 3:**
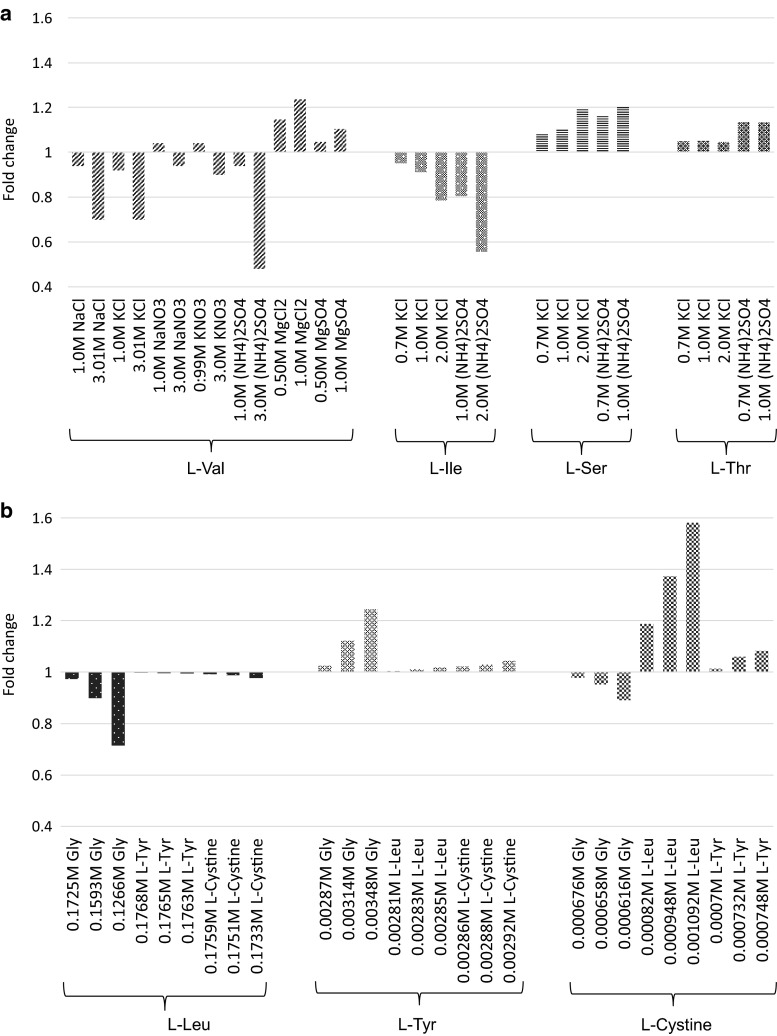
Effect of salts and amino acid on the solubility of amino acids in aqueous solution. The fold change of the solubility of amino acids in aqueous solutions of salts (**a**) and other amino acids (**b**) at 298 K is presented. (Carta 1999; Ferreira et al. 2009; Held et al. 2014; Tome et al. 2013)

The salting in and salting out effects of electrolytes present in mammalian culture media influence amino acid solubility (Fig. 3). The extent of the influence can be estimated by the calculation of the activity coefficient (Ferreira et al. 2007, 2009; Tome et al. 2013; Held et al. 2014). Molecular dynamics studies reveal that this influence is exerted by the formation of a complex to alter the hydration of amino acid molecules (Tome et al. 2013). However, amino acids also affect each other’s solubility to a greater magnitude at lower concentrations as compared to electrolytes (Fig. 3). In the examination of this phenomenon, mixtures of up to three amino acids in aqueous solution have been studied and modeled (Grosse Daldrup et al. 2010, 2011; Carta 1999).

### Complex formation with metal ions

**Fig. 4:**
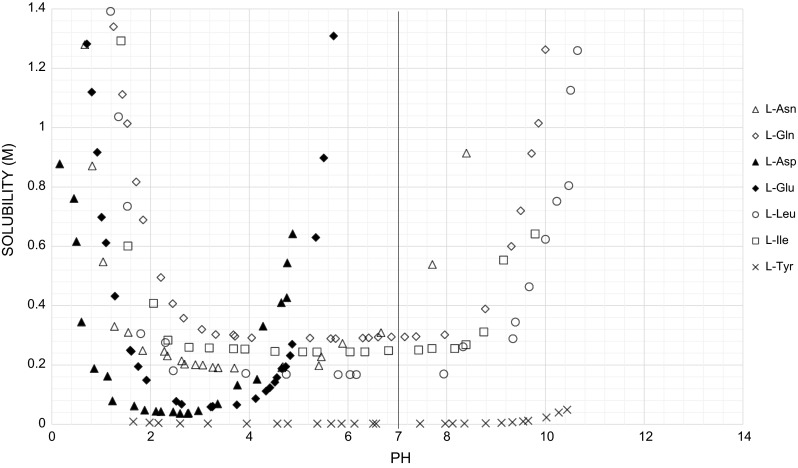
pH dependence of amino acid solubility. The solubility of an amino acid differs dependent on the amino acid and the pH conditions. Not all amino acids are zwitterions at neutral pH. Data from (Lee et al. 2013; Tseng et al. 2009)

Amino acids form complexes with alkali (Li, Na, K), alkali earth (Ca, Mg), and transition metals (Fe, Ni, Cu, Zn) most of which are present in CCM for mammalian cells. Monovalent, divalent, and trivalent (in the case of Fe^3+^) cations are capable of the formation of coordination modes through interactions with the nitrogen of the amino group, the hydroxyl oxygen of the carboxyl group, and the carbonyl oxygen of the carboxyl group of all amino acids (Jover et al. 2008). Other possible coordination modes depend on the amino acid side chain (Meng et al. 2012; Remko et al. 2010). Studies have been carried out to understand the nature of these interactions in solution and the influence of water molecules on these interactions (Remko and Rode 2006). Glycine has been extensively studied since the possible coordination modes it is capable of forming are limited (Jover et al. 2009; Armentrout et al. 2012; Marino et al. 2006; Constantino et al. 2005). These studies show that the size of the metal ion influences the type of complex formed with glycine and that the stability of the complexes formed are affected by hydration. The examination of l-histidine dimers shows that complexes formed with the cations influence the bonds of the imidozole group, the redox potential, and the p*K*_a_ value (Remko et al. 2010), which in turn influences other parameters, including the solubility of an amino acid (Fig. 4).

### Mixed crystal formation

Amino acids are capable of forming mixed crystals where the crystal lattice of one amino acid has the molecules of another amino acid embedded in it. This has been established for amino acids with similar side chains (l-Val, l-Leu, l-Ile, l-Ala) (Kamei et al. 2008; Kurosawa et al. 2005a, b; Yang et al. 2013), but the inclusion of l-Val, l-Leu, and l-Ile in crystals of l-Glu has also been demonstrated (Kitamura and Nakamura 2001). These amino acids are found at concentrations close to their solubility limit in media, feed, and supplement formulations, particularly for cells using the Glutamine synthase system (GS system) (Landauer 2014). Cell of the GS system are capable of synthesis of l-glutamine from l-glutamate. This is used as a selection mechanism with only l-glutamate being supplied to the cells. However, this could provide conditions for the crystallization of amino acids. The crystallization of amino acids would lower their availability to cells in culture and alter the composition of the cellular environment.

### Stability

l-Glutamine is instable in aqueous solutions and chemically decomposes to form the cyclic compound pyrolidonecarboxylic acid with the release of ammonia (Chen and Harcum 2006; Ha and Lee 2014; Purwaha et al. 2014). The auto-decomposition of l-glutamine leads to decreased availability in cellular processes. In small-scale cultures, l-glutamine is freshly prepared and added to media. In large-scale processes, stable l-glutamine dipeptides and dry powdered CCM address this degradation (van der Valk et al. 2010).

Although the degradation of l-asparagine and l-aspartate may not occur as free amino acids in solution, the degradation of these amino acid occur as residues in proteins (Wright 1990; Peters and Trout 2006). In proteins, l-asparagine residues are shown to be deaminated, whereas l-aspartate residues are liable to isomerization in a protein molecule. These deamination and isomerization reactions are pH dependent and are thus influenced by the environment in which the protein is present (Pace et al. 2013; Capasso et al. 1995).

Besides these reactions, amino acids in CCM are also liable to hydroxylation, nitration, nitrosylation, sulfoxidation, chlorination, and carboxylation (Stadtman and Levine 2003). These modified amino acids could be added to polypeptide chains by misrecognition by the aminoacyl tRNA-synthase enzyme and are hence of concern to peptide therapeutic cell culture processes (Popp et al. 2015).

## Considerations for the development of new mammalian CCM formulations

Owing to the varied nature of amino acids and their interaction with components of cell culture media, certain considerations have to be made in the development of CMM, especially in the case for large-scale bioprocesses where high titer and quality of the molecule of interest is desired. These considerations are:

1. The amino acid content of the cell culture environment should be in ratios dependent on the metabolism and metabolic rates of the cell clone used considering both proteogenic and non-proteogenic fates.
2. Transporters governed the intracellular availability of amino acids. The expression of these transporters and the factors capable of altering their functions should therefore be considered.
3. Since the solubility of amino acids as individual components differ from their solubility mixtures, the solubility of every new formulation should be evaluated when it is being developed and tested under its intended storage conditions.
4. Amino acids are capable of interacting with metal ions present in CCM, which in turn effect each other’s availability and stability.
5. The stability of amino acids are susceptible to changes in environmental conditions.
6. The dissolution of DPM should be considered to prevent loss of components during filtration.

## Conclusion and future prospects

The amino acids used in the cultivation of mammalian cells are particularly important, given the biological roles they occupy and the chemical properties they possess. Continuing to study the roles amino acids play in cell biology and scaled down bioprocesses leads to a better understanding of a cell’s specific culture conditions and might improve the yield and quality (for example, glycosylation) of bio therapeutics. A better understanding of the amino acid metabolism of cell clones helps prevent the build-up of metabolites that negatively influence mammalian cell culture processes (Rouiller et al. 2014; Duarte et al. 2014; Chen and Harcum 2006). Understanding the functioning of amino acid transporters would help provide amino acids to cells at desired intracellular locations while preventing imbalances.

Using the current understanding of chemical properties would help develop stable media and supplement formulations, with desired high concentrations of amino acids. There is however a need for the further understanding of the chemistry of amino acids in complex mixtures, particularly in the context of CCM. Little is known about the outcomes that components of CCM have on amino acid solubility. This understanding is particularly crucial for chemically defined CCM since amino acids are most often the component that limits the solubility of a CCM formulation. Knowledge in this area would help achieve higher concentration of amino acids in CCM that are currently being used (Landauer 2014; Grosse Daldrup et al. 2010, 2011). Considering the interactions that occur between amino acids and other components of media, particularly metal ions, would help further elucidate the biochemical roles of these interactions. The investigation of the stability of amino acids will help control the quality of the molecule of interest and highlight the parameters that need to be controlled (Pace et al. 2013). The use of DPM is one method used to address the stability of amino acids. However, with the use of DPM, a better understanding of amino acid dissolution kinetics in complex mixtures is needed to ensure the availability of all ingredients of a formulation to mammalian cells in culture.

Cells are known to interact with the extracellular milieu, but the dynamics of these interaction is only beginning to be understood (Banerjee 2012; Wang et al. 2015). Further research is required to better characterize these interactions and elucidate new ones. However, this first requires the understanding of chemical interactions that take place in mammalian CCM in the absence of cells. Chemical and biochemical knowledge in this area would provide insight into the nature of these molecules, their interactions, and the roles that they occupy in relation to mammalian cells. The impact of this research would be of benefit to fields including amino acid crystallization, drug protein interaction, drug dissolution, cell biology (Hart et al. 2015), and cancer biology (Labuschagne et al. 2014).

## Electronic supplementary material

Below is the link to the electronic supplementary material.

Supplementary material 1 (XLSX 66 kb)

Supplementary material 2 (XLSX 48 kb)

Supplementary material 3 (PDF 358 kb)

## Abbreviations

αKG: Alpha-ketoglutarate
CCM: Cell culture media
DMEM: Dulbecco’s modification of eagles medium
DPM: Dry powdered media
GS: Glutamine synthetase
SLC: Solute carrier
THF: Tetrahydrofolate
