# Supplementary material for: Amino acids in the cultivation of mammalian cells
Source: Amino Acids. 2016 Feb 1;48:1161–71. doi: 10.1007/s00726-016-2181-8 (PMC4833841; doi:10.1007/s00726-016-2181-8)
Supplement: Supplementary file 3 — Supplementary material 3 (PDF 358 kb) [file 726_2016_2181_MOESM3_ESM.pdf]

Amino acid	DMEM F12 AA composition [%](M)	CHO-K1 Proteome AA composition[%] (P)	Δ= M-P	T=M+P
Glycine	1.734240765	6.257339149	-4.5231	7.99158
L-Alanine	0.411593141	6.724959379	-6.31337	7.136553
L-Arginine	13.64269401	5.468423014	8.174271	19.11112
L-Asparagine	0.693696306	3.60087063	-2.90717	4.294567
L-Aspartic acid	0.615077391	4.835767723	-4.22069	5.450845
L-Cysteine	1.624174284	2.312336858	5.100039	9.724713
L-Cystine	2.894100988	0	0	0
L-Glutamic acid	0.67982238	4.739593179	-4.05977	5.419416
L-Glutamine	33.75988688	6.940930484	26.81896	40.70082
L-Histidine	2.911674628	2.664741353	0.246933	5.576416
L-Isoleucine	5.038085037	4.469974	0.568111	9.508059
L-Leucine	5.461702248	10.0998996	-4.6382	15.5616
L-Lysine	8.439971721	5.782813495	2.657158	14.22279
L-Methionine	1.594576575	2.323230562	-0.72865	3.917807
L-Phenylalanine	3.281645991	3.784012486	-0.50237	7.065658
L-Proline	1.595501503	5.965966746	-4.37047	7.561468
L-Hydroxyproline	0	0	0	0
L-Serine	2.42793707	8.384106997	-5.95617	10.81204
L-Threonine	4.94374234	5.463648472	-0.51991	10.40739
L-Thryptophan	0.834285424	1.217348657	-0.38306	2.051634
L-Tyrosine	4.261585441	2.728701982	1.532883	6.990287
L-Valine	4.888246635	6.235335234	-1.34709	11.12358
	100	100		

Mini-Review Journal:Amino Acids
Title: Amino acids in the cultivation of mammalian
Andrew Salazar[ab], Michael Keusgen[a], Jörg von Hagen[b]
[a]Institute of Pharmaceutical Chemistry, University of Marburg, 35032 Marburg, Germany
[b]Biopharm Materials & Technologies R&D, Merck Lifescience, 64293 Darmstadt, Germany

Corresponding author e-mail: andrew.salazar@external.merckgroup.com

(Δ/T)*100
-56.5983
-88.4652
42.77234
-67.6942
-77.4319
52.44411
0
-74.9116
65.89292
4.428172
5.975047
-29.8054
18.6824
-18.5985
-7.10997
-57.7992
0
-55.0883
-4.99555
-18.6711
21.92876
-12.1102
